# Supplementary material for: Rapid transport of deformation-tuned nanoparticles across biological hydrogels and cellular barriers
Source: Nat Commun. 2018 Jul 4;9:2607. doi: 10.1038/s41467-018-05061-3 (PMC6031689; doi:10.1038/s41467-018-05061-3)
Supplement: Supplementary file 1 — Supplementary Information [file 41467_2018_5061_MOESM1_ESM.pdf]

Supplementary Information

Yu et al. Rapid Transport of Deformation-Tuned Nanoparticles across Biological Hydrogels and Cellular Barriers.

Table of Contents

Supplementary Note 1. Page 4

Supplementary Note 2. Page 4

Supplementary Note 3. Page 5

Supplementary Note 4. Page 5

Supplementary Note 5. Page 5

Supplementary Note 6. Page 6

Supplementary Note 7. Page 7

Supplementary Note 8. Page 7

Supplementary Note 9. Page 8

Supplementary Note 10. Page 10

Supplementary Note 11. Page 11

Supplementary Note 12. Page 13

Supplementary Note 13. Page 13

Supplementary Figure 1. Page 15

Supplementary Figure 2. Page 16

Supplementary Figure 3. Page 17

Supplementary Figure 4. Page 18

Supplementary Figure 5. Page 19

Supplementary Figure 6. Page 20

Supplementary Figure 7.	Page 21
Supplementary Figure 8.	Page 22
Supplementary Figure 9.	Page 23
Supplementary Figure 10.	Page 24
Supplementary Figure 11.	Page 25
Supplementary Figure 12.	Page 26
Supplementary Figure 13.	Page 27
Supplementary Figure 14.	Page 28
Supplementary Table 1.	Page 29
Supplementary Table 2.	Page 30
Supplementary Table 3.	Page 31
Supplementary Table 4.	Page 32
Supplementary Table 5.	Page 33
Supplementary Table 6.	Page 34
Supplementary Table 7.	Page 35
Supplementary Table 8.	Page 36
Supplementary References.	Page 37

Supplementary Note 1. Stability of NPs in simulated gastrointestinal (GI) fluids

To investigate the stability of DiI-labeled NPs in simulated GI fluids, the DiI-labeled NPs were incubated in SGF and SIF. SGF and SIF were prepared according to U.S. Pharmacopeial Convention (USP) requirements. SGF was composed of 0.2% sodium chloride (NaCl) and 0.32% pepsin, and the pH was adjusted to 1.2 by concentrated HCl. SIF was composed of 0.68% monobasic potassium phosphate (KH2PO4) and 1% pancreatin, and the pH was adjusted to 6.8 by NaOH. Suspensions of NPs with different rigidities (1 mL at 1 mg/mL) were added to 4 mL of SIF or SGF and incubated for 6 h (SIF) or 2 h (SGF) at 37 °C with shaking at 100 rpm. At specified time points, samples were withdrawn and their fluorescence was determined. After incubation, all samples were centrifuged (4,000 rpm for 10 min), and the supernatants were collected for fluorescence detection. The fluorescence emission spectra of the NPs were determined. If the NPs were unstable or the core-shell structure was compromised, the labeled DiI would leak from the NPs, affecting the emitted fluorescence signal. As shown in Supplementary Fig. 1, the DiI signal intensity of the NPs in SGF and SIF did not vary over the incubation time. This result demonstrated that DiI did not leak from the system and that the NPs were stable and intact in the simulated digestive fluids.

Supplementary Note 2. Characterization of NPs and their diffusivity in PEO hydrogels and rat intestinal mucus

The poly(ethylene oxide) (PEO) hydrogels have been commonly used to simulate a biological hydrogel[1]. Next, we used multi-particle tracking (MPT) to investigate the movement of DiI-labeled NPs in PEO hydrogels. Liposomes without F127 were completely trapped in the PEO mesh structure, whereas those decorated with 5% F127 exhibited increased diffusivity in the hydrogel (Supplementary Fig. 2). Compared with the Lip-$F127_{5\%}$ NPs, the $PLGA_{50}$-Lip-$F127_{5\%}$, $PLGA_{110}$-Lip-$F127_{5\%}$, $PLGA_{130}$-Lip-$F127_{5\%}$, and $PLGA_{160}$-Lip-$F127_{5\%}$ NPs exhibited enhanced diffusivity, although the motion of these NPs remained constrained (Supplementary Fig. 2a). In

contrast, the $PLGA_{70}$-Lip-$F127_{5\%}$ and $PLGA_{90}$-Lip-$F127_{5\%}$ NPs diffused more readily in the hydrogels and displayed the largest diffusion areas. On a time scale of 1 s, the <MSD> value of the $PLGA_{70}$-Lip-$F127_{5\%}$ (semi-elastic) NPs was approximately 142.5-fold, 6.9-fold, and 6.4-fold higher than those of the Lip, Lip-$F127_{5\%}$ (soft), and $PLGA_{160}$-Lip-$F127_{5\%}$ NPs (hard) (Supplementary Table 1), respectively.

Supplementary Note 3. Mucus adhesive particle (MAP) transport in rat intestinal mucus *ex vivo*

Fluorescent, carboxylate-modified polystyrene NPs (PS-COOH) measuring 500 nm were purchased from Sigma and used as model mucoadhesive particles (MAPs) to confirm the barrier properties of the mucus gel. We used MPT to quantify the transport of the MAPs. The ensemble-averaged mean square displacement (<MSD>) of MAPs in rat intestinal mucus was dramatically reduced by more than 8,000-fold compared with the theoretical MSD of similarly sized NPs in water, indicating adhesive immobilization (Supplementary Fig. 3). The distribution of the individual particle MSD values was similar to data reported previously[2], indicating that the observations and conclusions in this study are free of potential biases.

Supplementary Note 4. Cellular uptake in cancer cells

Among soft, semi-elastic and hard NPs, the hard NPs showed higher red fluorescence intensities than the soft NPs and semi-elastic NPs. These results indicate that the NPs with different rigidities can be listed in order of decreasing cellular uptake capacity as follows: hard NPs, semi-elastic NPs and soft NPs. Moreover, Lip-80 showed higher red fluorescence intensities than Lip-200 NPs. Both Lip-80 and Lip-200 exhibited cellular uptake behavior superior to that of the soft NPs and semi-elastic NPs.

Supplementary Note 5. Distribution of NPs in rat intestinal mucus *ex vivo*.

For effective oral drug delivery, the uniform and deep distribution of NPs throughout the intestinal mucosa is beneficial[3]. We investigated how NP rigidity affects the mucus-penetrating capability of NPs in rat intestinal loops using Lip-F127$_{5\%}$ (soft), PLGA$_{70}$-Lip-F127$_{5\%}$ (semi-elastic), and PLGA$_{160}$-Lip-F127$_{5\%}$ (hard) NPs. After staining, the mucin fibers were incubated with ligated intestinal loops for 30 min, followed by fluorescence imaging. As shown in Supplementary Fig. 5a, both the soft and hard NPs exhibited a moderate level of coverage area in the mucus, whereas the semi-elastic NPs were uniformly and widely dispersed. Indeed, the coverage area of the semi-elastic NPs in mucus was approximately 3-fold greater than that of the soft and hard counterparts (Supplementary Fig. 5b). An orthogonal view of the mucus was also obtained, showing the amount and depth of NP diffusion within mucus (Supplementary Fig. 5c). For both the soft and hard NPs, a yellow layer corresponding to the colocalization of mucus and NPs was visible in the upper parts, demonstrating that a limited amount of these NPs diffused through the mucus. In contrast, the semi-elastic NPs almost completely filled the mucus, with deeper penetration than the soft and hard NPs along the *z*-direction. To further confirm these differences, we conducted co-incubations of red and green NPs with different rigidities in the same intestinal loops. Again, the semi-elastic NPs penetrated deeper than the soft and hard NPs, demonstrating the superior penetration capability of the semi-elastic NPs (Supplementary Fig. 5d).

Supplementary Note 6. Co-delivery of NPs with different fluorophores to rats

We used mixed NP suspensions to compare the penetration ability of the different types of NPs, avoiding the possibility of individual differences between rats. NPs with different fluorophores were mixed at equivalent concentrations. SD rats were then orally administered 1 mL of mixed NP suspensions and sacrificed after 2 h; small intestinal segments were harvested, sliced, and stained with DAPI for confocal

microscopy. Similar results proved that elasticity would affect the behavior of the particles *in vivo* and determine the fate of NPs-based mucosal drug delivery systems.

Supplementary Note 7. Comparison of the transport of semi-elastic NPs with liposomes of different sizes in mucus

It is recognized that decreasing the size of NPs is an effective way to facilitate mucus penetration. Herein, we synthesized NPs of different sizes (Lip-80 nm, Lip-120 nm, Lip-150 nm, and Lip-200 nm, which refer to liposomes with particle sizes of 80, 120, 150, and 200 nm, respectively) to compare the transport of NPs in mucus. As shown in Supplementary Fig. 8, the MSD of the NPs increased slightly as the particle size decreased. In contrast, after encapsulating the PLGA cores into liposomes, the MSD of the semi-NPs presented a substantial improvement, which indicated that elasticity might be a more important factor than size for the transport of liposomes across mucus.

Supplementary Note 8. Mucus penetrating particle (MPP) transport in rat intestinal mucus *ex vivo*

Inspired by pathogens that can freely transport through mucus, the Hanes Group has fabricated mucus penetrating particles (MPPs)[4]. By coating the particles with a high density of low-molecular-weight polyethylene glycol (PEG), these MPPs can overcome the mucus barrier efficiently and can provide sustained release, reside in the GI tract for longer time and reach target tissues. Herein, we fabricated two types of MPPs as reported previously for comparison. The PEGylated liposomes (DSPC-$PEG_{5\%}$) and PEGylated PLGA nanoparticles (PLGA-$PEG_{5\%}$) were synthesized according to previously described methods[5, 6]. MPT experiments were then conducted to observe the transport dynamics of fluorescently labeled NPs in mucus. As shown in Supplementary Fig. 9, the DSPC-$PEG_{5\%}$ and PLGA-$PEG_{5\%}$ NPs displayed excellent penetration in rat intestinal mucus; however, the semi-NPs

exhibited better diffusion behavior, which reflected the superiority of the semi-NPs as MPPs.

Supplementary Note 9. Simulation model and method

Similar to our previous work[7, 8], a regular polymer network was utilized to represent mucus fibers with a mesh size of 16σ, as shown in Supplementary Fig. 10. Each fiber was composed of a series of beads spanning the entire simulation box ($98\times98\times98\sigma^3$). Different fibers were cross-linked by a node bead to simulate entanglement and crosslink of mucin fibers. The bonded interaction energy between neighboring beads i and j in the polymer chain was described by a simple harmonic spring with a spring constant of $k_b = 23\varepsilon/\sigma^2$ and an equilibrium bond length of $r_0 = 2.0\sigma$

$$E_{bond} = \frac{1}{2}k_b\left(r - r_0\right)^2 \tag{1}$$

The energy constraining the bond angle was described by an equilibrium angle with a bending constant of $k_a = 4.6\varepsilon$ and the equilibrium bond angle of $\theta_0 = \pi$

$$E_{angle} = \frac{1}{2}k_a\left(\theta - \theta_0\right)^2 \tag{2}$$

During the simulations, polymer node beads were constrained by applying a spring to tether them to their initial positions. The spring constant was set to $k_{self} = 4.6\varepsilon/\sigma^2$. The Lennard-Jones (LJ) potential was used as follow to describe the non-bonded interactions $V\left(r_{ij}\right)$ between two beads

$$V\left(r_{ij}\right) = 4\varepsilon_{lj}\left[\left(\frac{b}{r_{ij}}\right)^{12} - \left(\frac{b}{r_{ij}}\right)^{6}\right] \qquad r_{ij} < r_c \tag{3}$$

where ε_{lj} was the depth of the energy well, b was the equilibrium length between two beads, and r_c was the cut-off distance. The interaction parameters were listed in Supplementary Table 5. Totally, the polymer network was constructed by 4860 beads, 5292 bonds and 5292 angles.

The NP with the size of 10σ was modelled using the one-particle-thick model[9] which captures NP elastic property, allowing for stiffness tuning of the NPs. Each type of NPs was composed of 501 beads. Following the notation from the original paper, the inter-particle interaction between each pair of NP beads was described by a combination of two functions, $u(r)$ and $\phi(\hat{\mathbf{r}}_{ij},\mathbf{n}_i,\mathbf{n}_j)$, which represented the distance and orientation dependences, respectively, as follows

$$U(\mathbf{r}_{ij},\mathbf{n}_i,\mathbf{n}_j)=\begin{cases} u_R(r)+\varepsilon_{beads-beads}\left[1-\phi(\hat{\mathbf{r}}_{ij},\mathbf{n}_i,\mathbf{n}_j)\right] & r<r_{\min} \\ u_A(r)\phi(\hat{\mathbf{r}}_{ij},\mathbf{n}_i,\mathbf{n}_j) & r_{\min}<r<r_c \end{cases} \tag{4}$$

where

$$u_A(r)=-\varepsilon_{beads-beads}\cos^{2\zeta}\left(\frac{\pi}{2}\frac{r-r_{\min}}{r_c-r_{\min}}\right) \qquad r_{\min}<r<r_c \tag{5}$$

$$u_R(r)=\varepsilon_{beads-beads}\left[\left(\frac{r_{\min}}{r}\right)^4-2\left(\frac{r_{\min}}{r}\right)^2\right] \qquad r<r_{\min} \tag{6}$$

$$\phi(\hat{\mathbf{r}}_{ij},\mathbf{n}_i,\mathbf{n}_j)=1+\mu\left(a(\hat{\mathbf{r}}_{ij},\mathbf{n}_i,\mathbf{n}_j)-1\right) \tag{7}$$

$$a(\hat{\mathbf{r}}_{ij},\mathbf{n}_i,\mathbf{n}_j)=(\mathbf{n}_i\times\hat{\mathbf{r}}_{ij})\cdot(\mathbf{n}_j\times\hat{\mathbf{r}}_{ij})+\sin\theta_0(\mathbf{n}_i-\mathbf{n}_j)\cdot\hat{\mathbf{r}}_{ij}-\sin^2\theta_0 \tag{8}$$

In these equations $\mathbf{r}_i$ and $\mathbf{r}_j$ represented the center position vectors of bead i and j, $\mathbf{r}_{ij}=\mathbf{r}_i-\mathbf{r}_j$, $r=\|\mathbf{r}_{ij}\|$ and $\hat{\mathbf{r}}_{ij}=\mathbf{r}_i/r$. The unit vectors $\mathbf{n}_i$ and $\mathbf{n}_j$ represented the axes of symmetry of the bead i and j, respectively. The exponent ζ tuned the slope of the attraction between two beads. The parameter θ_0 and μ were related to the spontaneous curvature and bending rigidity of the NPs. In the simulations, we chose the same parameters $\varepsilon_{beads-beads}=1\varepsilon$, $\xi=4$, and $r_c=2.6\sigma$ as used in the original paper. The other two parameters (μ, $\sin\theta_0$) were used to modify the bending rigidity of different type of NPs and were listed in Supplementary Table 6. The interaction potential between the NPs and polymer network and NPs-NPs was also described by the LJ potential. The interaction between different NPs is zero, and the interaction parameters were listed in Supplementary Table 5. The interaction parameter between

the NPs and polymer chain was determined from All-Atom simulation and the details listed in the follows.

Supplementary Note 10. Interaction parameter between NPs and polymer chains

In our coarse-grained simulations, the interaction parameter between NPs and polymer chains was determined based on the results of full-atom simulations. In our experiments, the liposomes were coated with F127 molecules. To obtain the interaction energy between NPs and mucin fibers, we constructed two model systems: (1) one lipid bilayer interacting with one mucin glycoprotein chain; and (2) one long -(OCH2CH2)n-OH coil interacting with one mucin glycoprotein chain. From the first model we could extract the affinity between the bare liposome and polymer, while from the second one we could extract the affinity between the F127-coated liposome and polymer. In the full-atom simulations, the mucin glycoprotein chain composed by 13 O-Glycans was built as descripted by Yamada et al.[10] and Kim et al.[11]. Each O-Glycans comprises 5 groups with sequence of DGalpb1-3DGlcpNAcb1-3DGalpb1-3DGalpb1-3DGalpNAcb1-OH. The F127 molecules were represented by long PEG chains. A lipid bilayer was used to represent the surface of a liposome. The force field topology files of lipid molecule was downloaded from the Tieleman laboratory[12] and the topology files of -(OCH2CH2)n-OH and O-Glycans molecule were downloaded from automated force field topology builder (ATB) site[13]. Consider our simulation system, the partial charges of O-Glycans molecule were modified to a restrained electrostatic potential (RESP) charge obtained from DFT calculations[14]. Simple-point charge (SPC) water molecules were added as provided by Gromacs package. Each system was initially placed in a simulation box with dimensions $6.52\times6.51\times10 nm^3$. An example of one of the systems, containing lipid membrane and mucin glycoprotein chain, is shown in Supplementary Fig. 11.

The model systems were energy-minimized using the steepest descent algorithm. Thereafter, the systems were equilibrated for 10 ns in the NPT ensemble, using the Nosé-Hoover thermostat[15] (with a reference temperature of 300 K and a coupling constant of 0.1 ps) together with the semi-isotropic Parrinello-Rahman barostat[16] (with a reference pressure of 1 atmosphere, a compressibility of $4.5\times10^{-5}\mathrm{bar}^{-1}$). Finally, a 20 ns run was performed in the NVT ensemble at 300K and interactions between mucin fiber and lipid bilayer/-(OCH2CH2)n-OH chain were calculated. In the simulations, all covalent bonds were constrained by the LINCS algorithm[17] with a time step of 1 fs. The Particle Mesh Ewald (PME) algorithm[18] was used for calculating the long-range electrostatic interactions. A distance cutoff of 1.1 nm was applied for the short-range nonbonded interactions. Periodic boundary conditions were used for all MD simulation systems. All the full-atom simulations, as well as the interaction energy analysis, were carried out with the GROMACS 5.1 software package[19].

After a serials of simulation, we found that the interaction energy between lipid bilayer and mucin fiber is about $-72.51kJ\cdot mol^{-1}\cdot nm^{-2}$, much larger than the interaction between mucin fiber and -(OCH2CH2)n-OH chains (about $-2.29kJ\cdot mol^{-1}\cdot nm^{-2}$). It means the interaction between the NPs and the mucus chain will decrease substantially when their surface is coated with F127, which is consistent with experimental descriptions. We then set the parameter $\varepsilon_{ij}=0.1$ in our coarse grained models, thus the interaction strength between the NPs and polymer chain is about $-5.17kJ\cdot mol^{-1}\cdot nm^{-2}$, close to the result from atomistic simulations ($-2.29kJ\cdot mol^{-1}\cdot nm^{-2}$). This justifies our choice of the interaction parameter $\varepsilon_{ij}=0.1$ between NPs and polymer chain in our coarse-grained simulation.

Supplementary Note 11. Effect of interaction parameters on the diffusion of NPs in a model system

In order to verify the robust of our simulation model, we modulated the interaction parameter between the polymer network and NPs, which represented the affinity between mucus and NPs, from $\varepsilon_{ij} = 0.02$ to $\varepsilon_{ij} = 0.2$ in the simulations (Supplementary Table 7). The results indicated that when the interaction between the NPs and polymer network is weak ($\varepsilon_{ij} = 0.02$), the diffusivity of the NPs will increase compare to the case of $\varepsilon_{ij} = 0.1$ (Fig. 6d-e and Supplementary Fig. 12a-b). Interestingly, in this situation the diffusion of the soft NPs is faster than those of the semi-elastic and hard NPs (Supplementary Fig. 12a-b).We attributed this to the low affinity that, without excessive deformation, the soft NPs could deform into ellipsoids as observed for semi-elastic NPs in polymers with a high affinity ($\varepsilon_{ij} = 0.1$).We examined the number of contacts between the beads of one NP and the polymers, as shown in Supplementary Fig. 12c. All types of NPs showed approximately 5 contacts with the polymers, indicating the low affinity between the NPs and polymers. With the increase of the interaction between the NPs and polymer network ($\varepsilon_{ij} = 0.05$), the diffusivity of the NPs decreased obviously (Supplementary Fig. 12d-e) due to the trap of the polymer network, although the contact between the NPs and polymer did not increase (Supplementary Fig. 12f). In addition, the semi-elastic NPs began to diffuse faster than the soft and hard NPs (Supplementary Fig. 12d-e). However, when the interaction was strong ($\varepsilon_{ij} = 0.2$), all the NPs attached themselves to the polymers tightly, and no obvious diffusion happened (Supplementary Fig. 12g-h). In this case, the number of contacts between the NP and the polymers network increased to 20 (Supplementary Fig. 12i), which was several fold higher than the cases with moderate affinity ($\varepsilon_{ij} = 0.1$ and $\varepsilon_{ij} = 0.05$) and low affinity ($\varepsilon_{ij} = 0.02$). These results suggest that the rigidity-tuned fast diffusion of NPs was actually dependent on the affinity of the polymer network.

Supplementary Note 12. Effect of the pore size of the polymer network

We also tuned the pore size of polymer network from 14σ to 18σ in the simulations to investigate the pore size effect on the diffusion of NPs. We kept all the interaction parameters as used in the main text and changed the pore size of the polymer network. The results showed that when the polymer grid shrink (14σ), none of the NPs could diffuse freely in the network (Supplementary Fig. 13). Indeed, when the pore size of the network decreased, or the size of NPs increased, the NPs would be trapped in the polymer network, which was consistent with our experimental observations (Supplementary Table 2). However, when the pore size of polymer network was large (18σ), the semi-elastic and the hard NPs would diffuse freely in the network (Supplementary Fig. 13). In this situation, the restriction of the polymer network to these NPs was impaired. While for the soft NPs, the diffusion was not notable enhanced as those for the semi-elastic and hard NPs (Supplementary Fig. 13). The reason was still that they would deformed excessively and attached themselves to the polymers as mentioned above.

Supplementary Note 13. The diffusivity of the rigid ellipsoids NPs in the polymer network

To conform the shape deform of the spherical semi-elastic NPs during the diffusion, some additional simulations were performed. In these simulations two types of rigid ellipsoidal NPs with aspect ratio (AR) 2:1 and 3:1 are investigated, respectively. The results indicated that the diffusivities of the rigid NPs (one spherical and two ellipsoidal NPs) were influenced by the aspect ratio of the NPs (Supplementary Fig. 14). The MSD values of the ellipsoidal NPs are higher than the spherical NPs. The calculated diffusivity of the ellipsoidal NPs with AR=3 and AR=2 was 3-fold and 2-fold higher than the spherical NPs. The diffusivity of the spherical semi-elastic NPs lied between those of the rigid spherical NPs and rigid ellipsoidal NPs with aspect ratio 2:1 (Supplementary Fig. 14). These result further demonstrated that the

semi-elastic NPs would change their shape from a sphere to an ellipsoids during diffusion. In conclusion, the fast diffusion of the semi-elastic NPs was actually dependent on the shape deform during interaction with the polymer network.

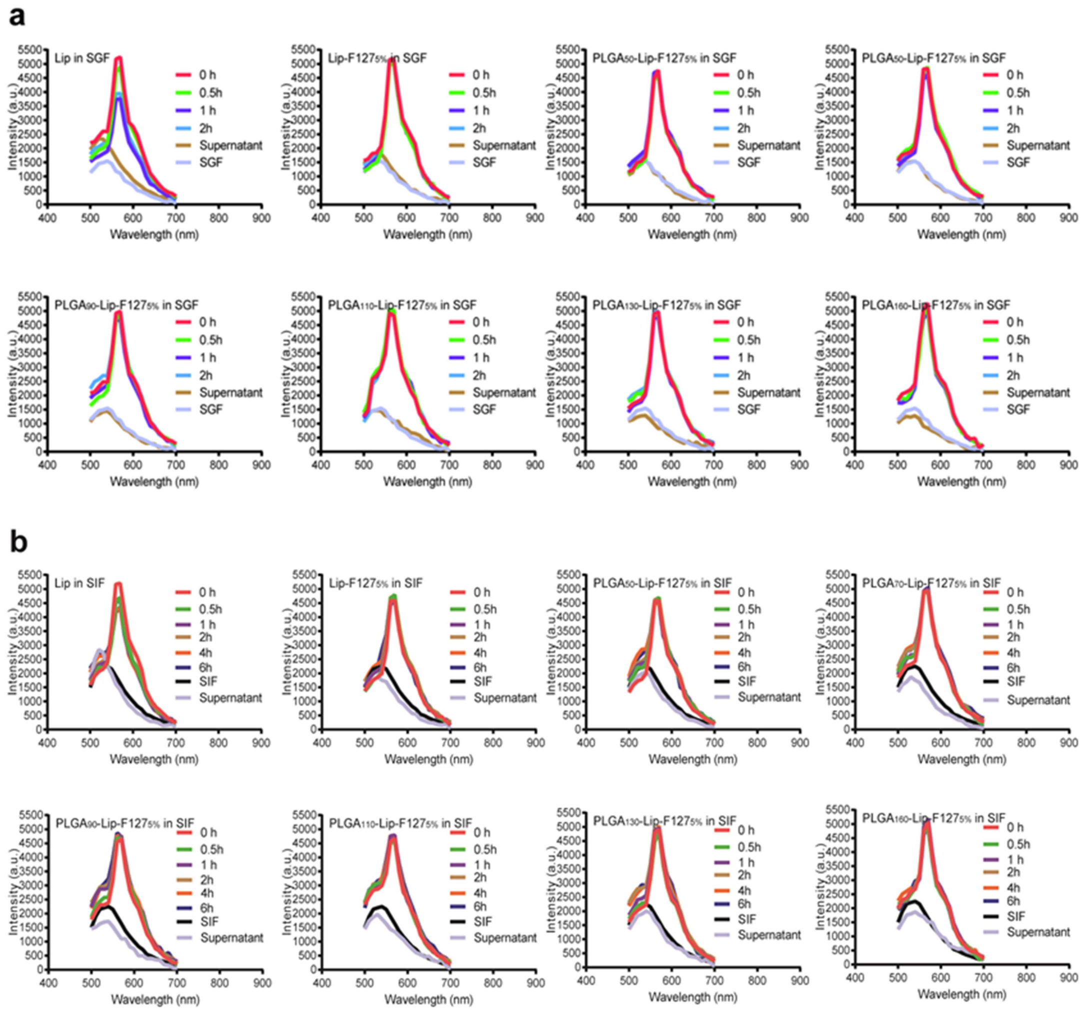

Supplementary Figure 1. Stability of NPs in simulated GI fluids. (a) The fluorescence emission spectra of DiI-labeled NPs incubated in SGF. (b) The fluorescence emission spectra of DiI-labeled NPs incubated in SIF.

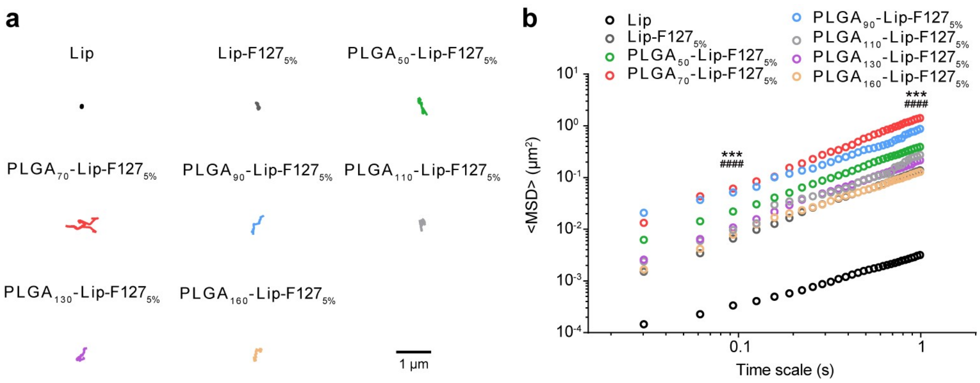

Supplementary Figure 2. Transport of NPs with various rigidities in simulated biological hydrogels. (a) Representative trajectories of particle motion in 1 s in PEO hydrogel. (b) <MSD> values as a function of time for particles in PEO hydrogel. *****P* < 0.0001, $PLGA_{70}$-Lip-$F127_{5\%}$ compared to Lip-$F127_{5\%}$, and $^{\#\#\#\#}$P < 0.0001, $PLGA_{70}$-Lip-$F127_{5\%}$ compared to $PLGA_{160}$-Lip-$F127_{5\%}$.

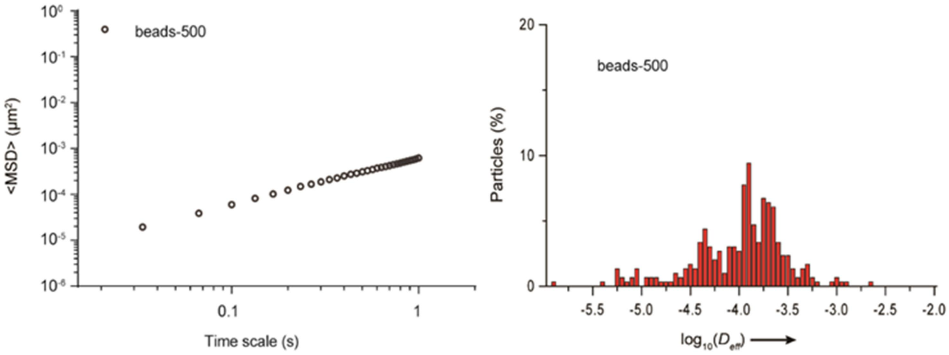

Supplementary Figure 3. Transport of MAPs in rat intestinal mucus. MSD and distributions of the logarithms of the effective diffusivity (D_{eff}) values of 500-nm beads.

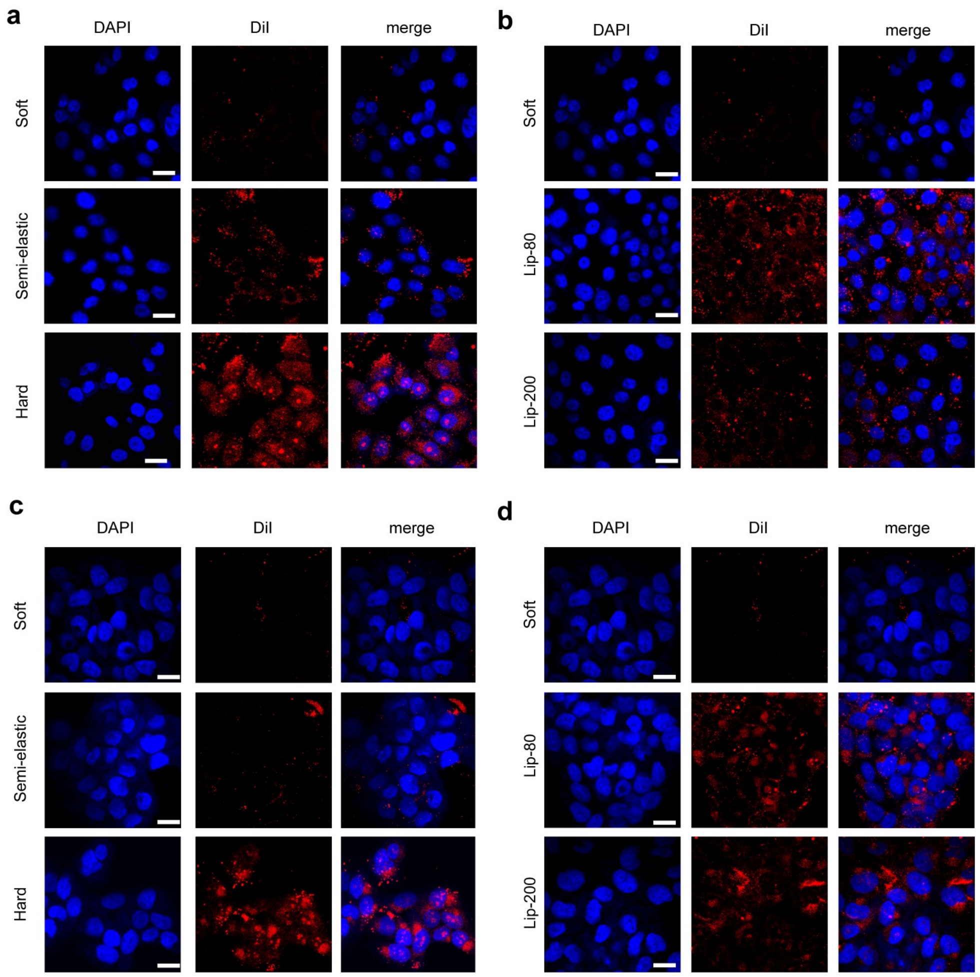

Supplementary Figure 4. Cellular uptake in cancer cells. BxPC-3 cells (a) and (b). HPSC cells (c) and (d). Blue: DAPI Red: DiI-labeled NPs. Scale bar: 20 μm.

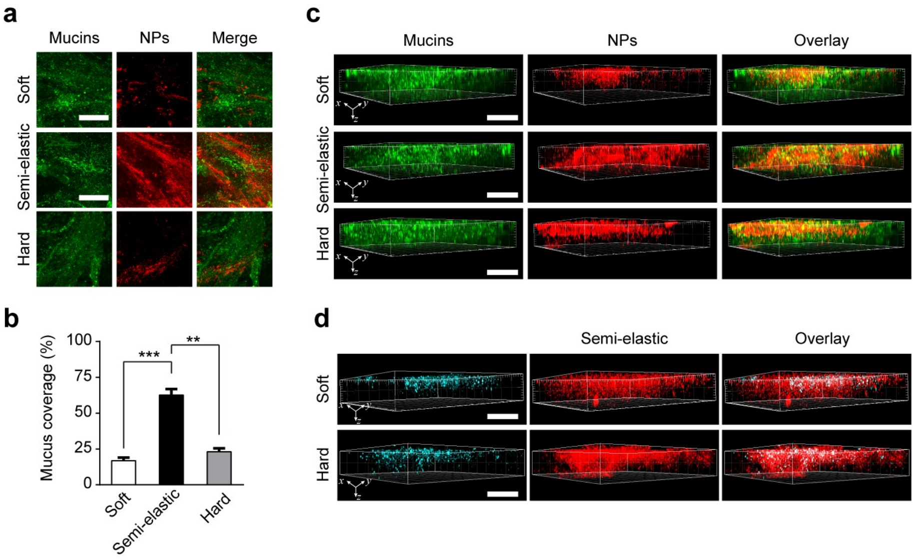

Supplementary Figure 5. Distribution of NPs in rat intestinal mucus *ex vivo*. (a) 2D coverage of NPs after diffusion in intestinal mucus. (**b**) Quantification of NP coverage in the mucus shown in (**a**). (**c**) 3D images of mucus penetration. Green: mucus stained with Alexa Fluor 488-WGA. Red: NPs. (**d**) A direct comparison of the penetration of semi-elastic NPs with the penetration of the softer and harder counterparts in the same mucus. Green: softer or harder NPs. Red: semi-elastic NPs. Images are representative of average values. Scale bar: 50 μm. Depth: 40 μm. Softer: Lip-$F127_{5\%}$ NPs; Semi: $PLGA_{70}$-Lip-$F127_{5\%}$ NPs; Harder: $PLGA_{160}$-Lip-$F127_{5\%}$ NPs. Data are shown as the means ± standard deviations (SDs). (n = 3) $^{****}P < 0.0001$, $PLGA_{70}$-Lip-$F127_{5\%}$ compared to Lip-$F127_{5\%}$, and $^{\#\#\#\#}P < 0.0001$, $PLGA_{70}$-Lip-$F127_{5\%}$ compared to $PLGA_{160}$-Lip-$F127_{5\%}$.

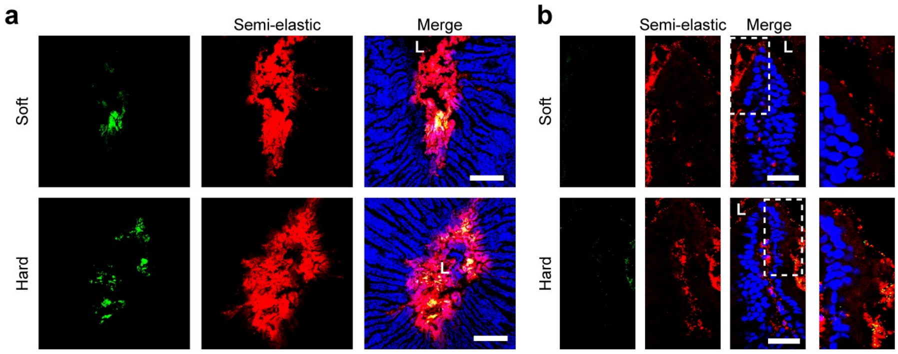

Supplementary Figure 6. Direct comparison of the distribution of NPs with different elasticities in the small intestine. (a) Distribution of NPs in the intestinal lumen. Scale bars: 200 μm. (b) Observation of a single villi at a higher magnification. Images are representative of the averages. Blue: nuclei of the intestinal villi. Green: soft or hard NPs. Red: semi-elastic NPs. Scale bars: 50 μm.

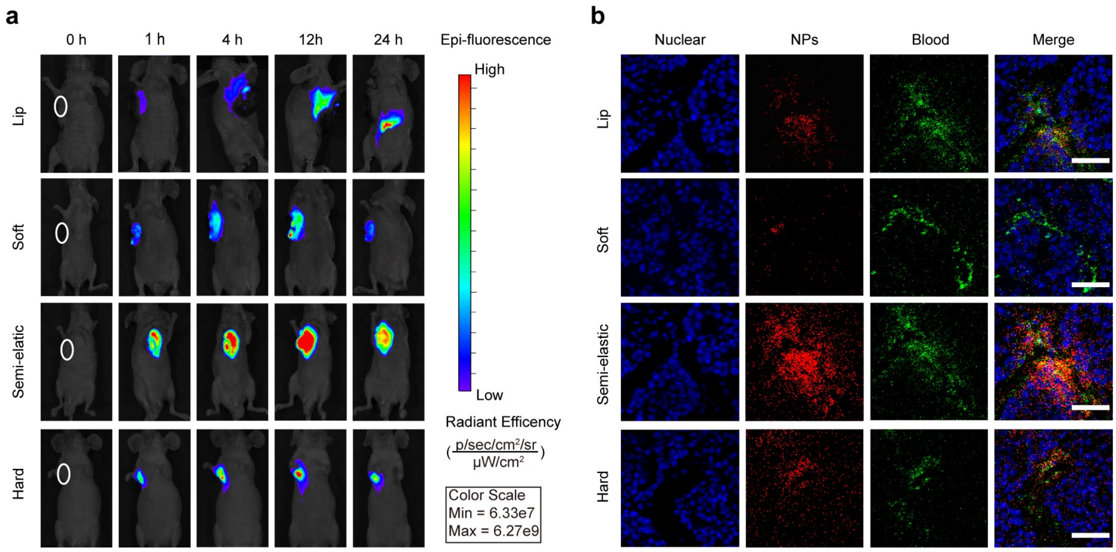

Supplementary Figure 7. (a) In vivo imaging of nude mice bearing BxPC-3 and HPSC xenografts administered IR783-labeled Lip, soft, semi-elastic, or hard NPs after injection (tumors are marked by a white circle). (b) Distribution of DiI-labeled softer, semi-elastic, and harder NPs in tumor slices of BxPC-3 & HPSC tumor xenografts 12 h after tail vein injection. The cell nuclei were counterstained with DAPI. The tumor vessels were labeled with anti-CD31 antibody. Scale bars: 50 μm. Softer: Lip-$F127_{5\%}$ NPs; Semi: $PLGA_{70}$-Lip-$F127_{5\%}$ NPs; Harder: $PLGA_{160}$-Lip-$F127_{5\%}$ NPs.

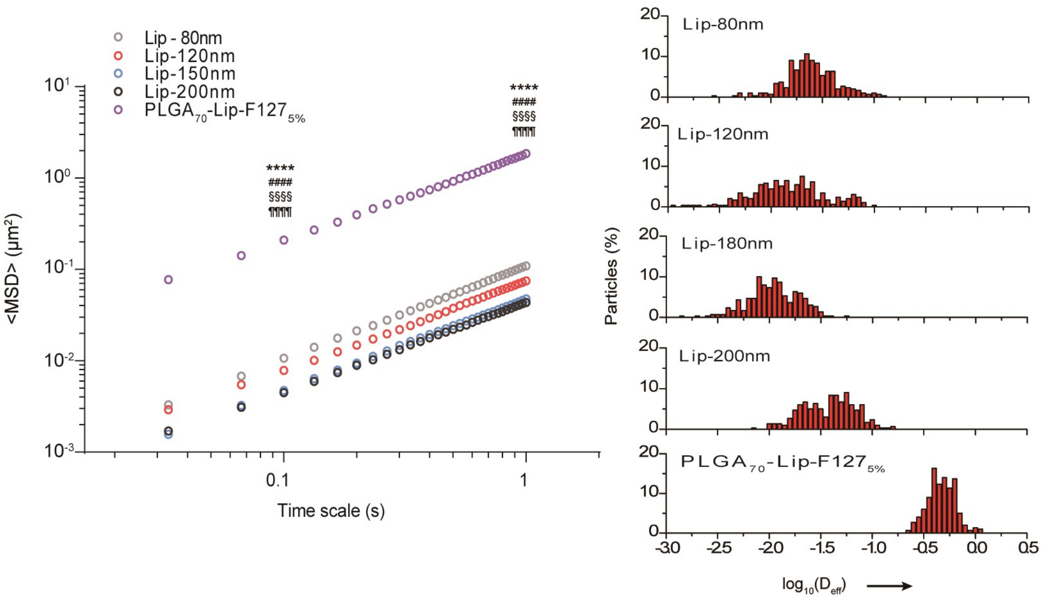

Supplementary Figure 8. Transport of NPs of different sizes (80 nm, 120 nm, 150 nm, and 200 nm) and semi-NPs in rat intestinal mucus. MSD and distributions of the logarithms of the effective diffusivities (D_{eff}) of nanoparticles of different sizes and semi-NPs. ****$P < 0.0001$, $PLGA_{70}$-Lip-$F127_{5\%}$ compared to Lip-80 nm, ####$P < 0.0001$, $PLGA_{70}$-Lip-$F127_{5\%}$ compared to Lip-120 nm, §§§§$P < 0.0001$, $PLGA_{70}$-Lip-$F127_{5\%}$ compared to Lip-150 nm, and ¶¶¶¶$P < 0.0001$, $PLGA_{70}$-Lip-$F127_{5\%}$ compared to Lip-200 nm.

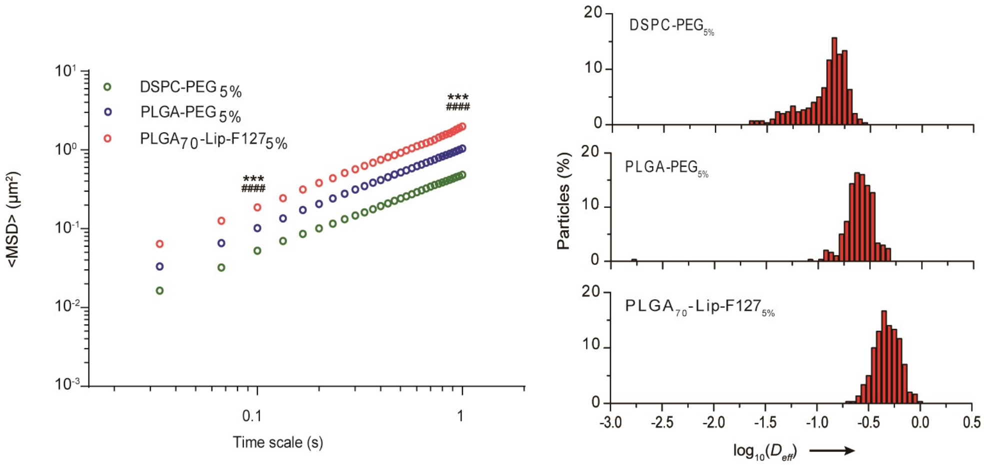

Supplementary Figure 9. Transport of different types of MPPs in rat intestinal mucus. MSD and distributions of the logarithms of the effective diffusivity (D_{eff}) values of different MPPs. ***$P < 0.001$, PLGA$_{70}$-Lip-F127$_{5\%}$ compared to PLGA-PEG$_{5\%}$, and $^{\#\#\#\#}P < 0.0001$, PLGA$_{70}$-Lip-F127$_{5\%}$ compared to DSPC-PEG$_{5\%}$.

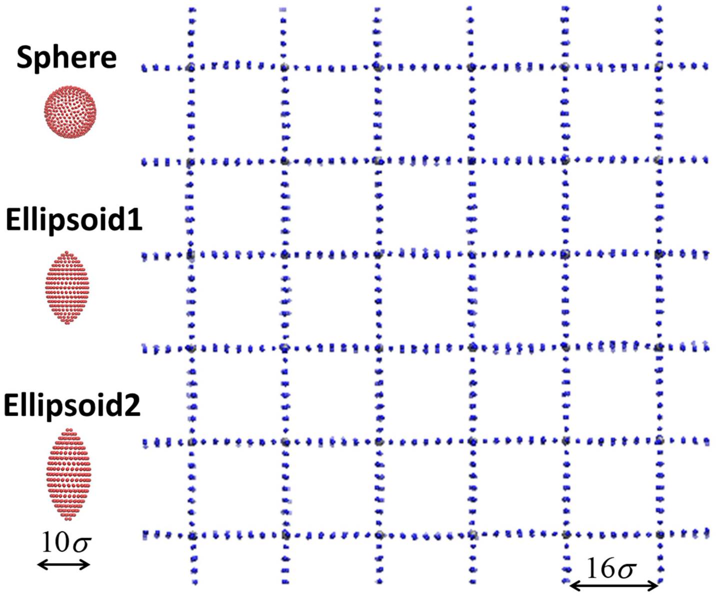

Supplementary Figure 10. The coarse grained models for diffusion of NPs in our simulations. The regular polymer network was utilized to represent mucus fibers with a mesh size of 16σ. The NPs are modelled using the one-particle-thick model. The diameter of the sphere NP is 10σ, and two rigid ellipsoids NPs with different aspect ratio (2 and 3) have the same volume as the sphere NP.

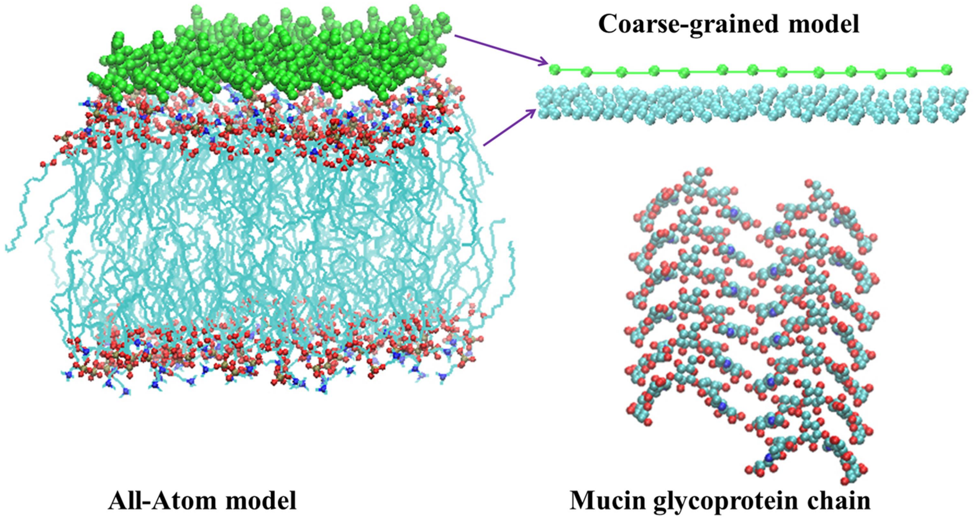

Supplementary Figure 11. A typical system of simulation. The All-Atom system contained a lipid membrane, a mucin glycoprotein chain above the membrane. The mucin glycoprotein chain was modeled as a combination of 13 O-Glycans. The coarse-grained system is composed of a lipid membrane and a long polymer chain above the lipid membrane (Cyan Beads: lipid membrane; Green beads: the polymer chain). In the coarse-grained model, a single bead of polymer chain and lipid membrane represents one O-Glycan and lipid molecule, respectively.

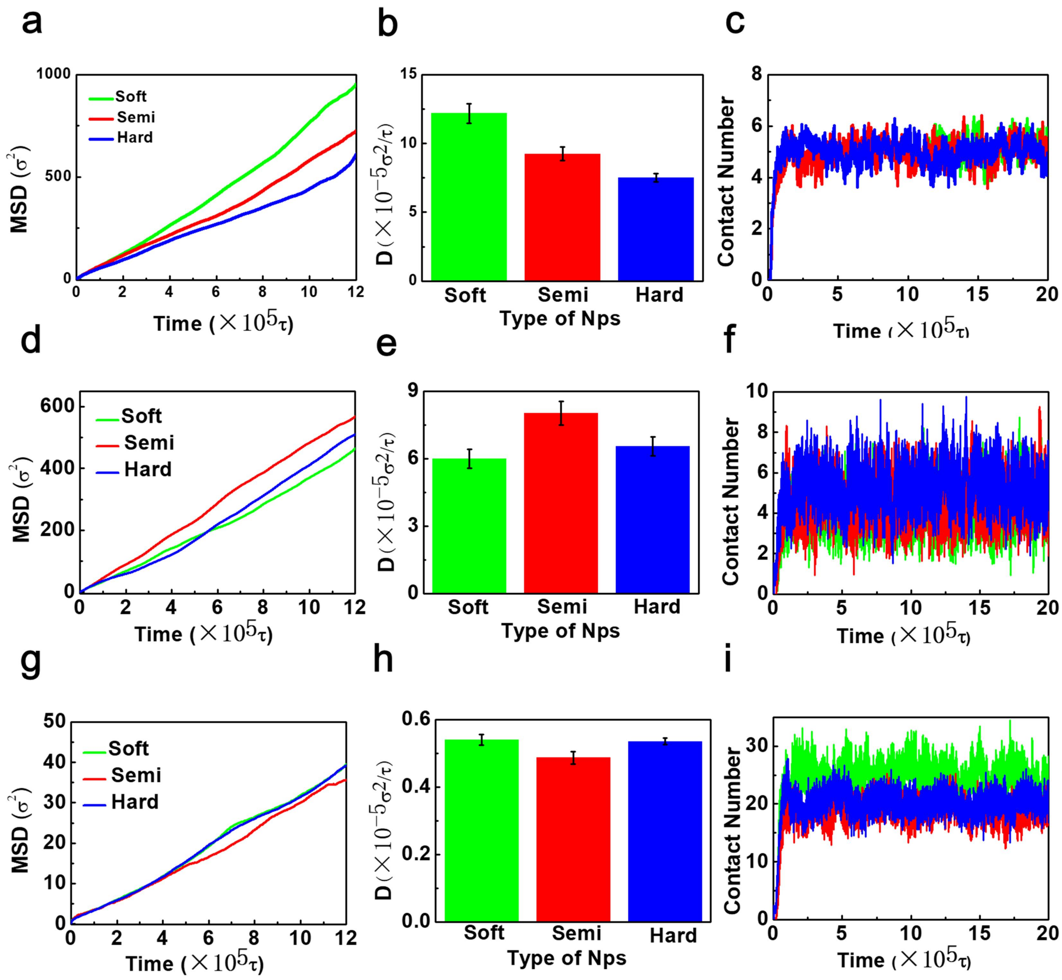

Supplementary Figure 12. Effect of interaction parameters on the diffusion of NPs in a model system. **(a)-(c)** The MSDs, diffusivities, and contact number for three types of NPs when the interaction parameter between polymer network and NPs decreases to $\varepsilon_{ij} = 0.02$. **(d)-(f)** The MSDs, diffusivities, and contact number for three types of NPs when the interaction parameter between polymer network and NPs increases to $\varepsilon_{ij} = 0.05$. **(g)-(i)** The MSDs, diffusivities, and contact number for three types of NPs when the interaction parameter between polymer network and NPs increases to $\varepsilon_{ij} = 0.2$. The green, red, and blue lines represent soft, semi-elastic, and hard NPs, respectively. Data are shown as the means ± standard deviations (SDs). (n = 3)

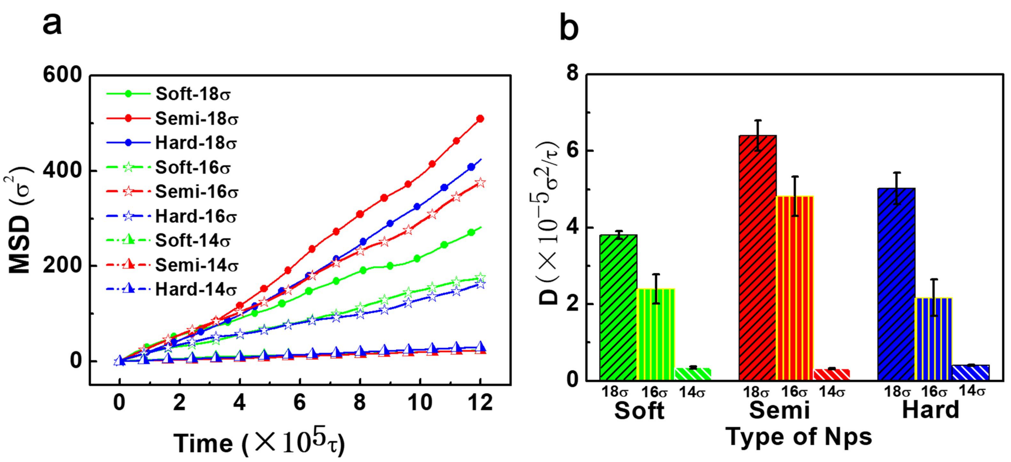

Supplementary Figure 13. Effect of the pore size of the polymer network on the diffusion of NPs in a model system. The **(a)** MSDs and **(b)** diffusivities for three types of NPs when the pore size of the polymer network is tuned from 14σ to 18σ gradually. The green, red, and blue lines represent soft, semi-elastic, and hard NPs, respectively. Data are shown as the means ± standard deviations (SDs). (n = 3)

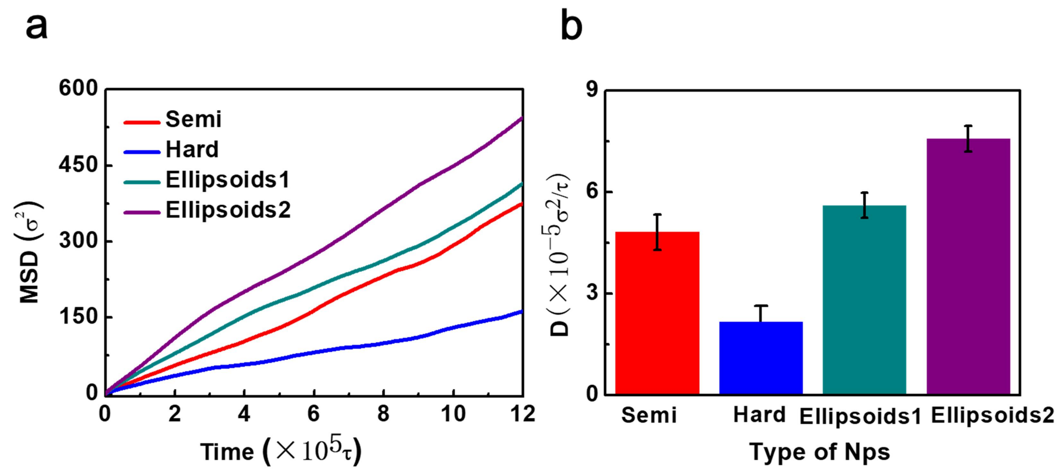

Supplementary Figure 14. The diffusivity of different types of NPs in the polymer network. **(a)** The MSDs for different types of NPs and **(b)** the diffusivities for different types of NPs. The red, blue, dark cyan, and purple lines represent semi-elastic NPs, hard spherical NPs, rigid ellipsoidal NPs with aspect ratio 2, and rigid ellipsoidal NPs with aspect ratio 3, respectively. Data are shown as the means ± standard deviations (SDs). (n = 3)

Supplementary Table 1. Characterization of NPs and their diffusivity in PEO hydrogels and rat intestinal mucus.

Nanoparticle	Hydrodynamic diameter (nm)	PDI	Zeta potential (mV)	Diffusivity in PEO hydrogel (μm²/s)	Diffusivity in mucus (μm²/s)
Lip	204.9±6.9	0.133	-11.3±1.9	0.004	0.019
Lip-$F127_{5\%}$	209.4±3.8	0.123	-5.8±1.9	0.084	0.168
$PLGA_{50}$-Lip-$F127_{5\%}$	196.7±3.8	0.154	-5.38±2.5	0.199	0.661
$PLGA_{70}$-Lip-$F127_{5\%}$	203.6±3.9	0.149	-4.8±0.6	0.57	1.852
$PLGA_{90}$-Lip-$F127_{5\%}$	200.5±5.2	0.164	-6.3±2.5	0.39	1.242
$PLGA_{110}$-Lip-$F127_{5\%}$	205.5±7.4	0.139	-5.99±2.78	0.15	0.427
$PLGA_{130}$-Lip-$F127_{5\%}$	213±3.2	0.142	-6.69±2.17	0.129	0.288
$PLGA_{160}$-Lip-$F127_{5\%}$	217.4±8.0	0.219	-4.3±1.8	0.089	0.218

Supplementary Table 2. Comparison of the mean diffusivity of liposomes of different sizes and semi-elastic NPs.

Nanoparticle	Hydrodynamic diameter (nm)	PDI	Zeta potential (mV)	Diffusivity ($\mu m^2/s$)	Ratio of diffusivity (NPs/liposome)
Lip-80 nm	84.0 ± 5.1	0.126	-4.5 ± 3.2	0.109	5.7
Lip-120 nm	117.4 ± 6.8	0.137	-7.7 ± 1.1	0.075	3.9
Lip-150 nm	151.8 ± 4.8	0.130	-5.2 ± 2.1	0.047	2.5
Lip-200 nm	204.9 ± 6.9	0.133	-11.3 ± 1.9	0.019	/
$PLGA_{70}$-Lip-$F127_{5\%}$	203.6 ± 3.9	0.149	-4.8 ± 0.6	1.851	97.4

Supplementary Table 3. Comparison of the mean diffusivity of different MPPs

Nanoparticle	Hydrodynamic diameter (nm)	PDI	Zeta potential (mV)	Diffusivity (μm^2/s)	Ratio of diffusivity (NPs/ $PLGA_{70}$-Lip-$F127_{5\%}$)
DSPC-$PEG_{5\%}$	197.9±4.2	0.101	-3.3±3.5	0.485	0.24
PLGA-$PEG_{5\%}$	200.2±1.5	0.156	-6.1±2.1	1.2671	0.64
$PLGA_{70}$-Lip-$F127_{5\%}$	201.8±8.6	0.178	-4.5±1.7	1.984	1.00

Supplementary Table 4. Pharmacokinetic parameters of Dox after oral administration of different Dox formulations at a dose of 10 mg/kg to rats. (n = 3)

Formulation	C_{max} (ng/mL)	T_{max} (h)	$AUC_{0\rightarrow\infty}$ （ug.h/L）	F_{rel}(%)
DOX	74.5±5.1	0.5	1,148±263	100
Soft	225.5±56.6	1	2,340±133	204
Semi	1,103.5±95.5	1	9,353±665	815
Hard	401.2±65.2	1	3,241±117	282

Supplementary Table 5. The LJ parameters used in simulations

type 1	type 2	$\varepsilon_{lj}(\varepsilon)$	$b(\sigma)$	$r_c(\sigma)$
NPs	NPs	0.01	1.0	2.0
Polymer	Polymer	0.1	1.0	2.0
NPs	Polymer	0.1	2.0	5.0

Supplementary Table 6. The different parameters used to tune the stiffness of three NPs (i.e., soft, semi, and hard NPs)

	μ	$\sin\theta_0$
Soft NPs	2.5	0.045
Semi NPs	8.0	0.045
Hard NPs	Move as a rigid sphere	

Supplementary Table 7. The LJ parameters used in simulations to study the effect of the affinity between NPs and polymer network

type 1	type 2	$\varepsilon_{lj}(\varepsilon)$	$b(\sigma)$	$r_c(\sigma)$
NPs	NPs	0.01	1.0	2.0
Polymer	Polymer	0.1	1.0	2.0
NPs	Polymer	0.02	2.0	5.0
		0.05	2.0	5.0
		0.2	2.0	5.0

Supplementary Table 8. The LJ parameters used in simulations for study the diffusivity of the rigid ellipsoids NP in the polymer network

type 1	type 2	$\varepsilon_{lj}(\varepsilon)$	$b(\sigma)$	$r_c(\sigma)$
NPs	NPs	0.01	1.0	2.0
Polymer	Polymer	0.1	1.0	2.0
NPs	Polymer	0.1	2.0	5.0

Supplementary References

1. Xue C, Zheng X, Chen K, Tian Y, Hu G. Probing non-gaussianity in confined diffusion of nanoparticles. *The journal of physical chemistry letters* **7**, 514-519 (2016).
2. Maisel K, Ensign L, Reddy M, Cone R, Hanes J. Effect of surface chemistry on nanoparticle interaction with gastrointestinal mucus and distribution in the gastrointestinal tract following oral and rectal administration in the mouse. *Journal of Controlled Release* **197**, 48-57 (2015).
3. Wu L, Shan W, Zhang Z, Huang Y. Engineering nanomaterials to overcome the mucosal barrier by modulating surface properties. *Advanced drug delivery reviews*, (2017).
4. Lai SK, Wang Y-Y, Hanes J. Mucus-penetrating nanoparticles for drug and gene delivery to mucosal tissues. *Advanced drug delivery reviews* **61**, 158-171 (2009).
5. Yu T, Chan KW, Anonuevo A, Song X, Schuster BS, Chattopadhyay S*, et al.* Liposome-based mucus-penetrating particles (MPP) for mucosal theranostics: demonstration of diamagnetic chemical exchange saturation transfer (diaCEST) magnetic resonance imaging (MRI). *Nanomedicine: Nanotechnology, Biology and Medicine* **11**, 401-405 (2015).
6. Xu Q, Ensign LM, Boylan NJ, Schön A, Gong X, Yang J-C*, et al.* Impact of surface polyethylene glycol (PEG) density on biodegradable nanoparticle transport in mucus ex vivo and distribution in vivo. *ACS nano* **9**, 9217-9227 (2015).
7. Yu M, Wang J, Yang Y, Zhu C, Su Q, Guo S*, et al.* Rotation-facilitated rapid transport of nanorods in mucosal tissues. *Nano letters* **16**, 7176-7182 (2016).
8. Wang J, Shi X. Molecular dynamics simulation of diffusion of nanoparticles in mucus. *Acta Mechanica Solida Sinica* **30**, 241-247 (2017).

9. Yuan H, Huang C, Li J, Lykotrafitis G, Zhang S. One-particle-thick, solvent-free, coarse-grained model for biological and biomimetic fluid membranes. *Physical Review E* **82**, 011905 (2010).
10. Yamada K, Hirabayashi J, Kakehi K. Analysis of O-glycans as 9-fluorenylmethyl derivatives and its application to the studies on glycan array. *Analytical chemistry* **85**, 3325-3333 (2013).
11. Kim YS, Gum J, Brockhausen I. Mucin glycoproteins in neoplasia. *Glycoconjugate journal* **13**, 693-707 (1996).
12. Peter Tieleman's Biocomputing Group. [*Internet*]. Available: http://wcm.ucalgary.ca/tieleman/downloads.
13. Malde AK, Zuo L, Breeze M, Stroet M, Poger D, Nair PC*, et al.* An automated force field topology builder (ATB) and repository: version 1.0. *Journal of chemical theory and computation* **7**, 4026-4037 (2011).
14. Bayly CI, Cieplak P, Cornell W, Kollman PA. A well-behaved electrostatic potential based method using charge restraints for deriving atomic charges: the RESP model. *The Journal of Physical Chemistry* **97**, 10269-10280 (1993).
15. Hoover WG. Canonical dynamics: equilibrium phase-space distributions. *Physical review A* **31**, 1695 (1985).
16. Parrinello M, Rahman A. Polymorphic transitions in single crystals: A new molecular dynamics method. *Journal of Applied physics* **52**, 7182-7190 (1981).
17. Hess B, Bekker H, Berendsen HJ, Fraaije JG. LINCS: a linear constraint solver for molecular simulations. *Journal of computational chemistry* **18**, 1463-1472 (1997).
18. Darden T, York D, Pedersen L. Particle mesh Ewald: An N· log (N) method for Ewald sums in large systems. *The Journal of chemical physics* **98**, 10089-10092 (1993).

19. Abraham MJ, Murtola T, Schulz R, Páll S, Smith JC, Hess B*, et al.* GROMACS: High performance molecular simulations through multi-level parallelism from laptops to supercomputers. *SoftwareX* **1**, 19-25 (2015).
