# Supplementary material for: Rapid transport of deformation-tuned nanoparticles across biological hydrogels and cellular barriers
Source: Nat Commun. 2018 Jul 4;9:2607. doi: 10.1038/s41467-018-05061-3 (PMC6031689; doi:10.1038/s41467-018-05061-3)
Supplement: Supplementary file 3 — Description of Additional Supplementary Files [file 41467_2018_5061_MOESM3_ESM.docx]

**Description of Additional Supplementary Files**

File Name: Supplementary Movie 1

Description: The 3D mobility and morphology of soft NPs in hydrogel captured by super-resolution microscopy.

File Name: Supplementary Movie 2

Description: The 3D mobility and morphology of semi-elastic NPs in hydrogel captured by super-resolution microscopy.

File Name: Supplementary Movie 3

Description: The 3D mobility and morphology of hard NPs in hydrogel captured by super-resolution microscopy.
